# Prevention of Adriamycin-induced hepatic and renal toxicity in male BALB/c mice by a nutrient mixture

**DOI:** 10.3892/etm.2014.1535

**Published:** 2014-02-11

**Authors:** M. WAHEED ROOMI, TATIANA KALINOVSKY, NUSRATH WAHEED ROOMI, MATTHIAS RATH, ALEKSANDRA NIEDZWIECKI

**Affiliations:** Dr. Rath Research Institute, Santa Clara, CA 95050, USA

**Keywords:** Adriamycin, nutrient mixture, hepatic toxicity, renal toxicity, BALB/c mice, blood urea nitrogen, uric acid, creatinine, alanine aminotransferase, aspartate aminotransferase, γ-glutamyl transferase

## Abstract

Adriamycin (ADR), an antineoplastic antibiotic used in cancer therapy, is associated with toxicity to vital organs with long-term use. A nutrient mixture (NM) has previously been shown to exhibit a broad spectrum of therapeutic properties. The aim of the present study was to determine whether the NM is useful for preventing ADR-induced hepatic and nephric toxicity. Six-week-old male BALB/c mice were divided into four groups of six animals each. Groups A and C were fed a regular diet for three weeks and groups B and D were fed a diet supplemented with 1% NM. After three weeks, the mice in groups C and D received 20 mg/kg body weight ADR intraperitoneally, while those in groups A and B received saline alone. Animals were sacrificed after 24 h, blood samples were collected and serum was obtained for clinical chemistry. Organs were also excised and weighed. Administration of ADR to group C (control diet) resulted in a marked increase in hepatic alanine aminotransferase, aspartate aminotransferase and γ-glutamyl transferase levels and renal blood urea nitrogen, creatinine and uric acid serum markers. However, in group D (NM 1% diet), the serum markers were comparable with the levels of group A and B. Therefore, the results indicate that NM has the potential to protect against ADR-induced hepatic and nephric damage.

## Introduction

Adriamycin (ADR; doxorubicin hydrochloride), is an anthracycline antibiotic that is clinically used as an antineoplastic agent. The clinical efficacy of ADR, particularly for long-term treatment, is limited by the induction of hepatic and cardiac toxicities that are frequently lethal (1). Specific studies have proposed that ADR-induced toxicity is possibly mediated by oxidative damage to cellular components, including membrane lipids in the plasma membranes and mitochondria (2). When the concentration of generated reactive oxygen species exceeds the antioxidant capability of the cell, cellular oxidative damage occurs. Oxygen-derived free radicals and lipid peroxidation play a critical role in the pathogenesis of various liver diseases, including hepatic fibrosis (3,4). Doxorubicin has been shown to cause an imbalance between free oxygen radicals and antioxidant enzymes, resulting in tissue injury (5,6). Doxorubicin induces toxic effects on the liver by increasing the levels of superoxide dismutase, catalase and glutathione peroxidase enzymes in liver tissue (7,8). The modulation of these mediators has been indicated to prevent doxorubicin-induced toxicities in various organs (9).

Diverse antioxidants have been shown to prevent ADR-induced hepatotoxicity in rats (10,11). A unique nutrient formulation (NM), containing primarily ascorbic acid, lysine, proline, *N*-acetyl cysteine and green tea extract, has previously been shown to exhibit a broad spectrum of pharmacological, therapeutic, cardiovascular and chemoprotective properties (12). In previous studies, it was found that NM significantly inhibited acetaminophen- and carbon tetrachloride-induced hepatic and renal damage (13,14).

In the present study, the *in vivo* effects of the NM diet were examined in mice treated with ADR, focusing on renal and hepatic enzyme levels.

## Materials and methods

### Materials

ADR powder, obtained from Sigma-Aldrich (St. Louis, MO, USA), was diluted in warm saline (pH 7.4) to 20 mg/ml. The stock solution of the NM was composed of the following in the quantities indicated: 700 mg vitamin C (as ascorbic acid and as Mg, Ca and palmitate ascorbate); 1,000 mg L-lysine; 750 mg L-proline; 500 mg L-arginine; 200 mg *N*-acetyl cysteine; 1,000 mg standardized green tea extract (80% polyphenol); 30 μg selenium; 2 mg copper; 1 mg manganese; and 50 mg quercetin.

### Animals

Male BALB/c mice free of murine viruses, bacteria and parasites and ~6 weeks of age on arrival, were purchased from Simonsen Laboratories (Gilroy, CA, USA). The mice were maintained in microisolator cages under pathogen-free conditions on a 12-h light/dark schedule for one week. Animals were cared for in accordance with the institutional guidelines for the care and use of experimental animals.

### Experimental design

Following one week of isolation, mice were divided into four groups (A–D) with six animals per group. Mice in groups A and C were fed a regular Purina mouse chow diet (Laboratory Rodent Diet 5001 from Purina Mills, LLC, purchased from Newco Distributing Inc., Rancho Cucamonga, CA, USA) for three weeks, while those in groups B and D were fed a regular mouse chow diet supplemented with 1% (w/w) NM during that period. During the study, the mice consumed, on the average, 4 g of their respective diets per day. Thus, the supplemented mice received ~20 mg NM per day. After three weeks, the mice in groups C and D received a single injection of 20 mg ADR/kg intraperitoneally, while those in groups A and B received saline alone. After 24 h, mice were sacrificed, blood samples were obtained by cardiac puncture, serum was collected for clinical chemistry and livers, kidneys, hearts and lungs were excised and weighed.

### Statistical analysis

Results are expressed as mean + SD for each group. Data was analyzed by independent sample t tests using MedCalc Software (Ostend, Belgium). P<0.05 was considered to indicate a statistically significant difference.

## Results

### Renal toxicity

ADR-induced renal toxicity in BALB/c male mice was measured by renal serum enzyme levels. These changes were protected by the NM 1% diet.

### Mean serum blood urea nitrogen (BUN)

**Figure 1:**
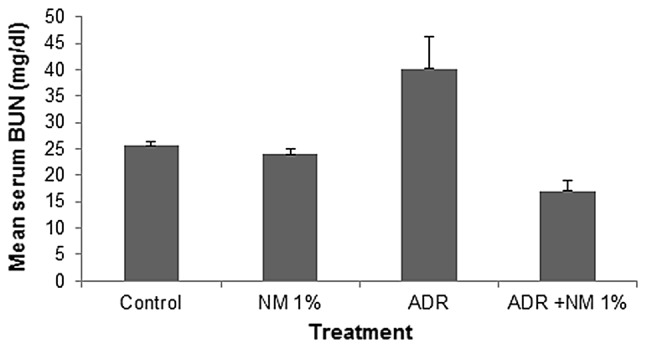
Effect of ADR and the NM 1% diet on mean serum BUN levels in male BALB/c mice. ADR, Adriamycin; NM, nutrient mixture; BUN, blood urea nitrogen.

In the untreated BALB/c mice that were fed the supplemented NM 1% diet, the mean serum BUN level was 93% of that in the control diet mice. In the mice fed the control diet, the administration of ADR increased the serum BUN level by 157% of the values in the saline-treated controls (P=0.0001). Of the BALB/c mice injected with ADR, the mice fed the NM 1% diet showed a mean serum BUN level that was 42% (P<0.0001) of that in mice fed the control diet. Furthermore, mice injected with ADR and fed NM 1% showed a 43.6% (P<0.001) reduction in mean serum BUN level compared with that in the control mice not injected with ADR (Fig. 1).

### Mean serum uric acid

**Figure 2:**
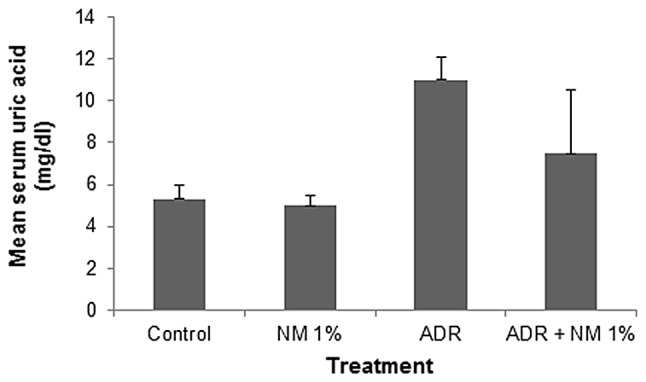
Effect of ADR and the NM 1% diet on mean serum uric acid levels in male BALB/c mice. ADR, Adriamycin; NM, nutrient mixture.

In the mice fed the control diet, the administration of ADR increased the mean serum uric acid level by 207% (P<0.0001) of that in the saline-treated mice. Of the BALB/c mice injected with ADR, the mice fed the NM 1% diet showed a mean serum uric acid level that was 68.2% (P=0.02) of that in mice fed the control diet. Mice injected with ADR and fed NM 1% showed no significant difference in mean serum uric acid level compared with those in the mice not injected with ADR on the control or NM 1% diets (Fig. 2).

### Mean serum creatinine

**Figure 3:**
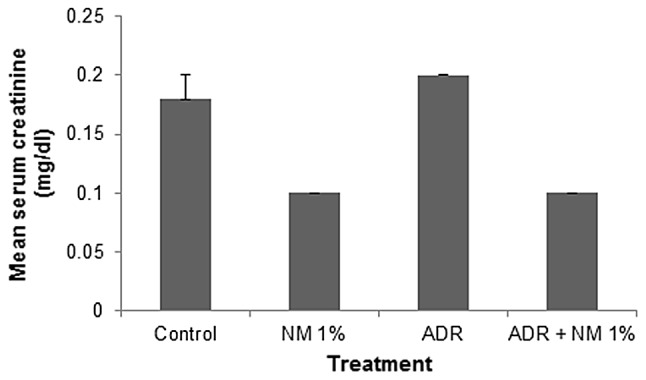
Effect of ADR and the NM 1% diet on mean serum creatinine levels in male BALB/c mice. ADR, Adriamycin; NM, nutrient mixture.

The mean serum creatinine level in untreated BALB/c mice fed the supplemented NM 1% diet was 56% (P<0.001) of that in control diet mice. In the mice fed the control diet, the administration of ADR increased the mean level of creatinine by 111% (P=0.03) of that in the saline-treated mice. Of the BALB/c mice injected with ADR, the mice fed the NM 1% diet showed a mean serum creatinine level that was 50% (P<0.0001) of that in mice fed the control diet. Furthermore, mice injected with ADR and fed NM 1% showed a 44.4% (P<0.0001) reduction in mean serum creatinine level compared with that in control diet mice not injected with ADR (Fig. 3).

### Hepatic toxicity

ADR-induced hepatic toxicity in BALB/c male mice was measured by hepatic serum enzyme levels. These changes were protected against by the NM 1% diet.

### Mean serum alanine aminotransferase (ALT)

**Figure 4:**
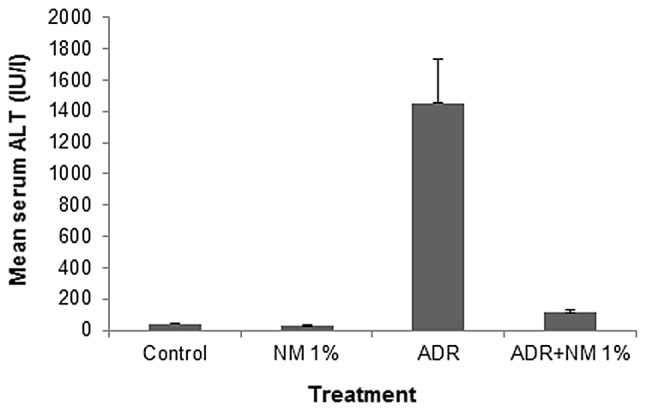
Effect of ADR and the NM 1% diet on mean serum ALT levels in male BALB/c mice. ADR, Adriamycin; NM, nutrient mixture; ALT, alanine aminotransferase.

Among the untreated BALB/c mice, mean serum ALT level decreased by 31% (P=0.004) following NM supplementation. In the mice fed the control diet, ADR treatment increased the ALT level by 3,718% (P<0.0001) of the level in the saline-treated control. Of the BALB/c mice injected with ADR, the mice fed the NM 1% diet showed a mean serum ALT level that was 7.9% (P<0.0001) of that in mice fed the control diet. Mice injected with ADR and fed NM 1% showed a mean serum ALT level that was 292% (P<0.001) of that shown by control mice not injected with ADR (Fig. 4).

### Mean serum aspartate aminotransferase (AST)

**Figure 5:**
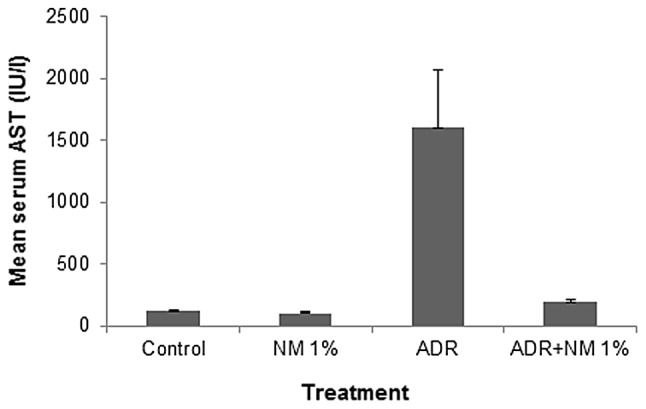
Effect of ADR and the NM 1% diet on mean serum AST levels in male BALB/c mice. ADR, Adriamycin; NM, nutrient mixture; AST, aspartate aminotransferase.

Among the untreated BALB/c mice, the mean serum AST level decreased by 12% (P=0.03) with NM supplementation. In the mice fed the control diet, the administration of ADR increased the mean serum AST level by 1,334% (P<0.0001) of the value in the saline-treated controls. Of the BALB/c mice injected with ADR, the mice fed the NM 1% diet showed a mean serum ALT level that was 12% (P<0.0001) of that in mice fed the control diet. Mice injected with ADR and fed NM 1% showed a mean serum AST level that was 163% (P<0.0001) of that shown by control mice not injected with ADR (Fig. 5).

### Mean serum γ-glutamyl transferase (γ-GT)

**Figure 6:**
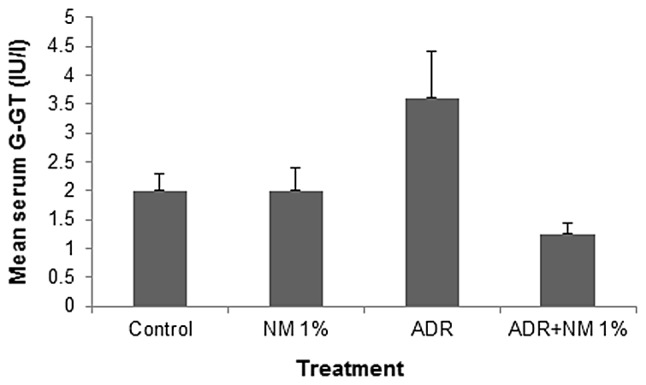
Effect of ADR and the NM 1% diet on mean serum γ-GT levels in male BALB/c mice. ADR, Adriamycin; NM, nutrient mixture; γ-GT, γ-glutamyltransferase.

In the mice fed the control diet, the mean serum γ-GT level increased by 180% (P=0.001) of the value in the saline-treated controls. Of the BALB/c mice injected with ADR, the mice fed the NM 1% diet showed a mean serum γ-GT level that was 34.7% (P<0.0001) of that in mice fed the control diet. Mice injected with ADR and fed NM 1% showed a mean serum γ-GT level that was 62.5% (P=0.0005) of that shown by control mice not injected with ADR. No significant difference was identified between the γ-GT levels of untreated mice in the two diet groups (Fig. 6).

### Vital organ weights

ADR injection into BALB/c mice did not have a significant effect on the weights of vital organs, as shown in Table I.

### Final body weights of mice

ADR injection into BALB/c mice reduced the mean final weight of the mice fed the control diet by 14.5% (P=0.0001) and the mice fed the NM 1% diet by 5% (P=0.04). In the mice not treated with ADR, the mean final weights of the control diet and NM 1% diet mice did not significantly differ. The mean initial weight of all the mice was 24.1±1.4 g, whereas the mean final weights for the untreated groups were 26.2±0.7 g for control diet mice and 26.4±1.4 g for the NM 1% diet mice. For the ADR-treated groups, the mean final weights were 22.4±1.2 g for control diet mice and 24.9±1.2 g for NM 1% diet mice (data not shown).

### Dietary intake

The mean dietary consumption by the mice in the two groups of mice fed the NM 1% diet (3.5±0.5 g) was 83.3% (P=0.036) of that consumed by the mice in the control diet groups (4.2±0.5 g) (data not shown).

## Discussion

The results of the present study demonstrate that pretreatment for three weeks with a diet supplemented with NM 1% reduced hepatic and renal damage in male BALB/c mice injected with a toxic dose (20 mg/kg body weight) of ADR. ADR treatment caused marked increases in the levels of hepatic serum markers, AST, ALT and γ-GT, in non-supplemented mice. Supplementation with NM retained the AST, ALT and γ-GT levels at normal levels. Elevated ALT and AST levels reflect hepatocellular inflammation, damage and necrosis, as additional AST and ALT are released into the bloodstream when a body tissue or organ, including the heart or liver, is diseased or damaged. The amount of AST in the blood is directly associated with the extent of tissue damage. Increased levels of γ-GT are associated with early liver cell damage or cholestatic disease. These elevated levels of serum indices for hepatocellular damage have been previously reported in a doxycycline-induced hepatoxicity model (15).

The ADR-treated mice also showed significantly increased levels of renal markers, including creatinine, uric acid and BUN. NM 1% dietary supplementation attenuated the increases in renal serum marker levels, to provide almost normal levels. The BUN/creatinine ratio is useful for the differential diagnosis of acute or chronic renal disease. Reduced renal perfusion, congestive heart failure or recent onset of urinary tract obstruction is likely to result in an increase in the BUN/creatinine ratio. The BUN/creatinine ratio for the untreated control BALB/c mice was 142, whereas when the mice were treated with ADR, the ratio increased to 200. NM 1% dietary pretreatment in BALB/c mice injected with ADR reduced the mean BUN/creatinine ratio to 170, a reduction of 15% compared with that observed in mice receiving the control diet prior to ADR administration.

Various antioxidants have been shown to prevent ADR-induced toxicity *in vivo*. A previous review by Grandados-Principal *et al* provided new evidence for the chemoprevention of doxorubicin toxicity using natural antioxidants, including vitamin E, vitamin C, coenzyme Q, carotenoids, vitamin A, flavonoids, polyphenol, resveratrol, antioxidants from virgin olive oil and selenium. The study offered new insights into the molecular mechanisms of doxorubicin toxicity with respect to DNA damage, free radicals and other parameters (16). The NM tested was formulated based on targeting various physiological processes involved in a wide spectrum of pathological conditions at the cellular level.

The antioxidant, vitamin C, was shown to protect against doxorubicin-induced cardiotoxicity and prolong the lives of mice and guinea pigs without interfering with the anticancer function of the drug (17). An additional component of the NM that is important for protecting the liver against toxicity is *N*-acetyl cysteine. This is used as an antidote for acetaminophen toxicity, as it increases glutathione stores, providing a glutathione substitute and directly conjugates with N-acetyl-*p*-benzoquinoneimine (NAPQI), a toxic metabolic by-product. *N*-Acetyl cysteine has been shown to protect animals from the cardiotoxicity of doxorubicin (18).

Green tea polyphenols have also shown protective effects against the administration of toxic chemicals. Pretreatment with epigallocatechin gallate (EGCG) led to a dose-dependent reduction of all the histological and biochemical variables of liver injury observed in carbon tetrachloride-treated mice (19). Green tea polyphenols reduced the severity of liver injury with lower concentrations of lipid peroxidation and proinflammatory nitric oxide generated mediators. Hasegawa *et al* reported that pretreatment of male rats with green tea as drinking water provided effective protection against the induction of hepatic degenerative changes by the carcinogen 2-nitropropane (20). Patil *et al* observed that green tea extract protected rats from doxorubicin-induced electrocardiographic changes and changes in biochemical markers, including lactate dehydrogenase, creatine kinase and glutamic oxaloacetate transaminase in serum, as well as superoxide dismutase, catalase and reduced glutathione, membrane bound enzymes and decreased lipid peroxidation in heart tissue (21).

Based on previously published studies, we hypothesize that metabolic effects are likely to result from the synergy of ascorbic acid, lysine, proline, green tea extract, arginine, *N*-acetyl cysteine, quercetin, selenium, copper and manganese. Combining these micronutrients expands metabolic targets, maximizing biological impact with lower doses of components. A previous study of the comparative effects of NM, green tea extract and EGCG on the inhibition of MMP-2 and MMP-9 secretion in various cancer cell lines with varying MMP secretion patterns, revealed the superior potency of NM over green tea extract and EGCG at equivalent doses (22).

In conclusion, the present study demonstrated that pretreatment with a diet supplemented with 1% NM for three weeks reduced hepatic and renal damage in BALB/c mice following the administration of a toxic dose of ADR. Supplementation with dietary NM reduced the ADR-induced elevated hepatic and renal serum markers in mice. Although clinical studies are required, the results obtained indicate the therapeutic potential of using NM adjunctively with ADR to protect against ADR-induced liver and kidney damage.

## Figures and Tables

**Table I:** Effect of ADR and the NM 1% diet on the mean weight of vital organs in BALB/c mice.

	Organ weight (g)			
	
Organ	Control diet	NM 1% diet	ADR + control diet	ADR + NM 1% diet
Liver	1.4±0.10	1.43±0.11	1.21±0.14	1.18±0.05
Kidney	0.41±0.04	0.47±0.05	0.37±0.07	0.41±0.03
Lung	0.14±0.02	0.15±0.22	0.14±0.01	0.14±0.02
Heart	0.12±0.15	0.13±0.01	0.10±0.01	0.12±0.01
Spleen	0.11±0.04	0.09±0.004	0.06±0.04	0.06±0.01

ADR, Adriamycin; NM, nutrient mixture.
